# Methionine and choline deficiency rewires transcriptional programs to recapitulate molecular features of human MASH

**DOI:** 10.1016/j.jlr.2026.101022

**Published:** 2026-03-18

**Authors:** Wenjing You, Jianfei Ji, Nicole Nguyen, Lauren Esp, Xiaoli Sun

**Affiliations:** 1 Department of Pharmacology, The University of Texas Health Science Center at San Antonio, San Antonio, TX, USA; 2 Department of Biochemistry and Structural Biology, The University of Texas Health Science Center at San Antonio, San Antonio, TX, USA; 3 Transplant Center, The University of Texas Health Science Center at San Antonio, San Antonio, TX, USA

**Keywords:** methionine and choline deficiency, MASH, transcriptional regulation, inflammation, lipid metabolism, stress response

## Abstract

Metabolic dysfunction-associated steatohepatitis (MASH) is a leading cause of cirrhosis and liver-related mortality, but it remains unclear how nutrient stresses drive coordinated transcriptional remodeling in the pathogenesis of MASH. Clinical studies reported that methionine and choline deficiency (MCD) promotes chronic liver diseases. Multiple types of MCD diets have been adopted to establish MASH mouse models. However, how MCD modulates cell-intrinsic transcriptional responses across parenchymal and nonparenchymal liver cell types, and whether these effects recapitulate human MASH, remains unclear. Here, we generated a customized MCD cell culture medium to induce nutrient stress in HepG2 cells, endothelial cells, bone marrow-derived macrophages, and hepatic stellate cells. RNA-Seq was performed to characterize transcriptional regulations in response to MCD. Across cell types, lack of methionine and choline induced transcriptional program of inflammatory and stress response and suppressed metabolic pathways and cell-cycle progression, suggesting a proliferation pause as a compensatory stress-adaptive response that preserves cell viability and essential functions. In addition to these shared responses, MCD stress also caused distinct cell type-specific outputs that could contribute to the pathogenesis of MASH. Integrated analysis of these datasets with human MASH liver single-nucleus transcriptomic data demonstrated that th MCD condition recapitulates multiple pathophysiological features of human MASH, including the elevated inflammation, enhanced hepatocyte death, disrupted redox balance, altered metabolic homeostasis, and hepatic stellate cell activation. These findings not only uncover how MCD stress promotes MASH progression but also provide a conceptual basis to guide future use of MCD diet-induced models in MASH studies.

Metabolic dysfunction-associated steatohepatitis (MASH) represents the progressive and inflammatory type of metabolic dysfunction-associated steatotic liver disease (1, 2, 3, 4, 5). It is defined by hepatic steatosis, inflammation, and liver injury that can culminate in fibrosis, cirrhosis, and hepatocellular carcinoma (6, 7, 8, 9, 10). Recent epidemiological studies highlight the increasing global burden of MASH (11, 12). An extensive body of work demonstrated that MASH pathogenesis is not dictated by hepatocytes alone but instead emerges from coordinated stress responses across multiple hepatic cell populations. In MASH, hepatocytes exhibit metabolic dysregulation and enhanced cell damage, whereas macrophages impair immune homeostasis, endothelial cells (ECs) reshape vascular and immune signaling, and hepatic stellate cells (HSCs) promote extracellular matrix (ECM) remodeling and fibrogenesis (13, 14). Human single-cell and single-nucleus transcriptomic studies have been used to characterize MASH-associated changes of cellular states in patient liver, providing a high-resolution view of the cellular composition, functional states, and gene expression programs in MASH (15, 16, 17). However, a persistent challenge is to delineate cell type-specific effects of disease-associated nutrient deficiency and understand how cell type-specific response contributes to the complex tissue remodeling in MASH.

Methionine and choline deficiency (MCD) provides a defined nutrient stress model that directly disrupts one-carbon metabolism and membrane phospholipid homeostasis (18). It impairs the methionine cycle and directly reduces cellular pools of S-adenosylmethionine (SAM), the principal methyl donor that links methylation capacity to chromatin regulation, redox balance, and lipid homeostasis pathways (18, 19, 20). Accumulating evidence from human fatty liver disease supports the clinical significance of one-carbon flux and SAM metabolism (21). Studies have shown that deficiency in hepatic SAM and related metabolites can promote lipid accumulation, inflammation, and progression toward steatohepatitis and fibrosis, suggesting that SAM is not only a biochemical readout of nutrient status but also a critical regulator shaping disease outcomes (20, 21, 22, 23). In preclinical research, different types of MCD diets, including the MCD diet, choline-deficient diet, and low methionine choline-deficient amino acid-defined high-fat diet, have been widely used to establish mouse MASH models (7, 24, 25, 26, 27, 28, 29, 30, 31, 32, 33, 34, 35). However, how MCD stress transcriptionally reprograms the cell populations involved in MASH pathogenesis remains unknown.

Here, we establish a transcriptomic dataset to define cellular responses to the MCD condition across four key cell types representing parenchymal and nonparenchymal compartments, using representative cell lines, including HepG2 (hepatocytes), human ECs, immortalized bone marrow-derived macrophages (iBMDMs), and human HSCs. This dataset enables direct comparison of shared and cell type-specific responses to MCD stress. Across all cell types, MCD induced a conserved transcriptional program characterized by activation of inflammatory and stress pathways, prominently involving interferon signaling, TNF-NF-κB, and interleukin-6 (IL6)-Janus kinase (JAK)-signal transducer and activator of transcription 3 (STAT3) signaling, and broad suppression of metabolic and proliferative processes. In parallel, each cell type exhibits a distinct functional change consistent with its specialized role. Comparative analysis with single-nucleus transcriptomic data from human MASH liver suggests that MCD recapitulates multiple pathophysiological features of human MASH, including elevated inflammation, increased hepatocyte death, disrupted redox balance, and HSC activation.

## Materials and Methods

### Cell culture

All cell types were maintained at 37°C in a humidified incubator with 5% CO_2_. HepG2 cells (ATCC, HB-8065) were maintained in high-glucose DMEM supplemented with 10% FBS and 1% penicillin/streptomycin (P/S). Human ECs (TeloHAEC; ATCC CRL-4052) were maintained in vascular basal medium (ATC PCS-100-030) supplemented with vascular EC growth kit-vascular endothelial growth factor (VEGF) (ATCC PCS-100-041) and 1% P/S. iBMDMs (36) were cultured in DMEM containing 10% heat-inactivated FBS and 1% P/S. Immortalized human HSCs (37) were maintained in high-glucose DMEM supplemented with 10% FBS and 1% P/S. For MCD treatment, cells were washed with PBS and then incubated in either control medium (high-glucose DMEM) or methionine and CD high-glucose DMEM for the indicated duration. All treatments were performed under serum-free conditions.

### MCD treatment

Cells were treated for 24 h with either high-glucose DMEM (Gibco 11995) as a control or a custom MCD medium manufactured by Life Technologies (Thermo Fisher Scientific) formulated based on the control DMEM but lacking methionine and choline. The MCD medium contained the following components (mg/l): phenol red (15), glycine (30), L-arginine hydrochloride (84), L-cystine·2HCl (63), L-glutamine (584), L-histidine hydrochloride-H_2_O (42), L-isoleucine (105), L-leucine (105), L-lysine hydrochloride (146), L-phenylalanine (66), L-serine (42), L-threonine (95), L-tryptophan (16), L-tyrosine disodium salt dihydrate (104), L-valine (94), D-calcium pantothenate (4), folic acid (4), niacinamide (4), pyridoxine hydrochloride (4), riboflavin (0.4), thiamine hydrochloride (4), i-inositol (7.2), calcium chloride (CaCl_2_) (anhydrous, 200), ferric nitrate (Fe(NO_3_)_3_·9H_2_O) (0.1), magnesium sulfate (MgSO_4_) (anhydrous, 97.67), potassium chloride (KCl) (400), sodium chloride (NaCl) (6,400), sodium phosphate monobasic (NaH_2_PO_4_·H_2_O) (125), D-glucose (dextrose, 4,500), and sodium pyruvate (110).

### RNA-Seq library preparation

Total RNA was extracted from cells using TRIzol reagent and purified using Quick RNA mini prep columns and RNase-free DNase digestion according to the manufacturer’s instructions (Life Technologies). RNA quality was measured by a Tapestation system. Sequencing libraries were prepared in biological replicates from polyA-enriched mRNA. RNA-Seq libraries were prepared from poly(A)-enriched mRNA as previously described (3, 8, 38, 39). Libraries were size selected and purified by Speedbeads, quantified using a Qubit dsDNA HS Assay Kit (Thermo Fisher Scientific), and sequenced on a Nextseq 2000 (Illumina) according to the manufacturer’s instructions.

### RNA-Seq analysis

RNA-Seq analysis was performed as previously described (8, 40). FASTQ files from sequencing experiments were mapped to hg38 for human and mm10 for mouse cells, using consistent gene annotation versions for each species. STAR with default parameters was used to map RNA-Seq experiments (41). To compare differential gene expression between indicated groups, HOMER’s analyzeRepeats with the option rna and the parameters -condenseGenes, -noadj, and -count exons was used (42). Each sequencing experiment was normalized to a total of 10^7^ uniquely mapped tags by adjusting the number of tags at each position in the genome to the correct fractional amount given the total tags mapped. Sequence experiments were visualized by preparing custom tracks for the UCSC Genome Browser. Differential gene expression was assessed with DESeq2 using HOMER’s getDiffExpression.pl with the parameters -*P*-adj 0.05 and -log_2_ fold 0.585 (for 1.5-fold differently expressed genes) (43). For all genes, the transcripts per million (TPM) values were plotted and colored according to fold change. For various ontology analyses, HOMER, Metascape, and Kyoto Encyclopedia of Genes and Genomes (KEGG) were used (44). The enrichGO and enricher functions in the clusterProfiler (45, 46) package in R were used for enrichment analysis.

### Gene set enrichment analysis

Gene set enrichment analysis (GSEA) was performed using the GSEA function in the clusterProfiler (46) package. For each cell type, preranked GSEA was conducted on the comparison between control and MCD medium conditions to identify significantly enriched pathways. Gene sets from the Hallmark, Reactome, and Gene Ontology (GO) biological processes were obtained from the Molecular Signatures Database using species-matched annotations. Enrichment results are presented as normalized enrichment scores (NESs) with associated significant values. Representative pathways are shown using standard GSEA enrichment plots, and significantly enriched gene sets across cell types are summarized by heatmaps of NES.

### Human single-nuclei RNA-Seq analysis

Publicly available human liver transcriptomic datasets were obtained from the Gene Expression Omnibus (GEO). Single-nucleus RNA-Seq (snRNA-Seq) data were retrieved from GEO (accession GSE244832), comprising 14 human liver samples, including MASH (n = 9) and normal livers (n = 5). Raw gene count matrices were processed and analyzed using Seurat v5. Quality control was performed to exclude low-quality nuclei and potential doublets. Nuclei with low gene complexity, excessive unique molecular identifier counts, or high mitochondrial transcript fractions were filtered to remove and were applied consistently across all samples. After filtration, gene expression counts were normalized using SCTransform (or log normalization as specified), and highly variable genes were identified. Dimensionality reduction was performed by principal component analysis, followed by Uniform Manifold Approximation and Projection (UMAP) for visualization. To correct for intersample batch effects, datasets were integrated using Seurat’s standard integration workflows. Cell-type annotations provided in the original study were retained and, where necessary, validated using canonical marker genes.

To assess the in vivo relevance of MCD-induced transcriptional programs, cell type-specific MCD gene signatures were defined from bulk RNA-Seq differential expression analysis. Gene sets were constructed from the top 100 upregulated and downregulated genes ranked by log2 fold change and adjusted *P* value, after filtering for robust expression (supplemental Table S1). Signature enrichment in snRNA-Seq data was quantified for robust expression using the Seurat AddModuleScore function. For each cell type, module scores were compared between MASH and healthy samples, and results were visualized on UMAP embeddings and as violin plots. Representative gene expression patterns were visualized using dot plots, where dot size represents the proportion of expressing nuclei, and color intensity reflects average scaled expression.

### Statistical analysis

For bulk RNA-Seq, differential expression between control and MCD conditions was assessed within each cell type using DESeq2, implemented through HOMER (getDiffExpression.pl), with the default Wald test framework. *P* values were adjusted for multiple testing using the Benjamini-Hochberg false discovery rate method. Genes were considered differentially expressed at an adjusted *P* < 0.05 and an absolute log_2_ fold change ≥0.585 (corresponding to ≥1.5-fold change), unless otherwise specified. Adjusted *P* values reported for individual genes derive from the same DESeq2 analysis. For visualization purposes, transcript abundance was expressed as TPM. TPM values were normalized to the mean of the control group for plotting and are presented as mean ± SEM across biological replicates.

For human snRNA-Seq analyses, statistical comparisons of module scores or gene expression levels between healthy and MASH groups were performed using the Wilcoxon rank-sum test implemented in Seurat. *P* values were adjusted for multiple testing using the Benjamini-Hochberg method within each panel of comparisons. For boxplots, center lines indicate the median, box boundaries represent the interquartile range, and whiskers extend to 1.5 times the interquartile range unless otherwise noted. Violin plots display the full distribution of single-nucleus values.

## Results

### MCD induces global transcriptional remodeling across different cell types

**Fig. 1:**
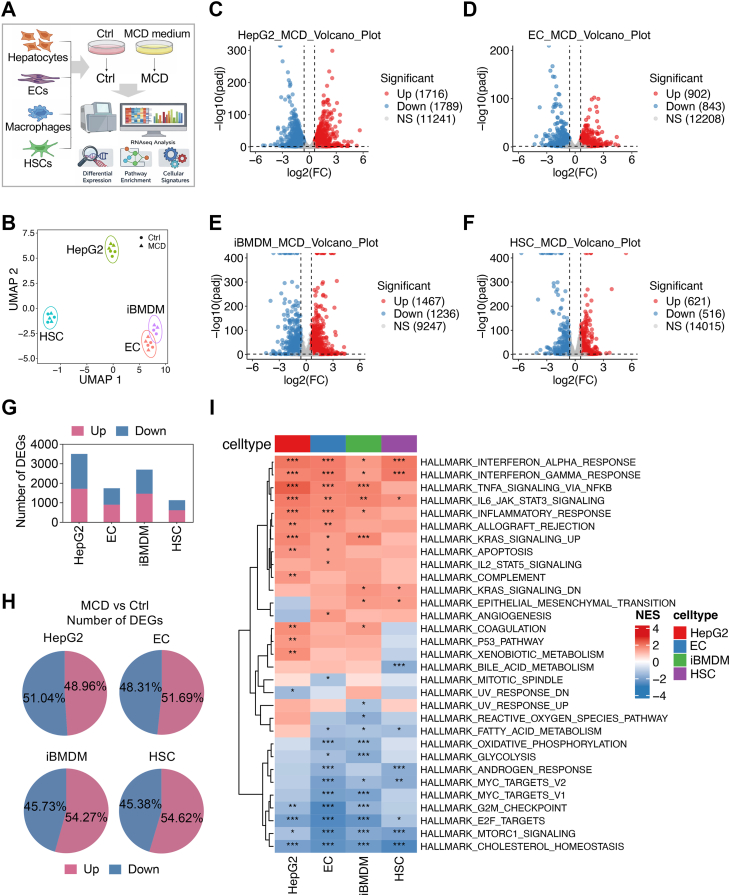
MCD induces global transcriptional remodeling across different cell types. A: Schematic overview of the experimental design. HepG2 cells, ECs, iBMDMs, and HSCs were cultured in control or MCD medium followed by bulk RNA-Seq and cross-cell type integrative transcriptomic analysis. B–I: Genes with a mean expression of at least three normalized tags in at least one condition were included in downstream analyses. B: UMAP projection of global transcriptomes across cell types. C–F: Volcano plots showing differential gene expression (MCD vs. Ctrl) in HepG2 cells (C), ECs (D), iBMDMs (E), and HSCs (F). Differentially expressed genes (DEGs) were defined by fold change >1.5 and P-adj <0.05. G: Total number of DEGs identified in each cell type. H: Proportion of upregulated and downregulated DEGs across cell types. I: Hallmark GSEA across the four cell types. Heatmap shows NESs for significantly enriched pathways (P-adj <0.05), highlighting conserved biological programs enriched in at least three cell types in response to MCD.

To determine how MCD reprograms the transcriptomic profile across cell types implicated in steatohepatitis and fibrogenesis, we performed RNA-Seq in HepG2 cells (hepatocyte model), ECs, iBMDMs (macrophages), and HSCs treated with control or MCD medium (Fig. 1A). Unsupervised analysis of global transcriptomes showed that cell identity accounted for the primary source of variation, with clear separation among HepG2, ECs, iBMDMs, and HSCs (Fig. 1B). Within each cell type, MCD induced a robust shift in global gene expression profiles, as evidenced by the clear segregation of control and MCD-treated samples in principal component analysis (supplemental Fig. S1A–D), indicating a robust response. Differential expression analysis demonstrated a pronounced and cell type-dependent transcriptional response to MCD. HepG2 exhibited the most robust response (fold change >1.5, p-adj <0.05), with 1,716 genes upregulated and 1,789 downregulated (Fig. 1C). iBMDMs showed similar extensive reprogramming (1,467 upregulated, 1,236 downregulated; Fig. 1D), followed by ECs (902 upregulated, 843 downregulated; Fig. 1E). HSCs displayed a comparatively smaller, yet substantial transcriptomic shift (621 upregulated, 516 downregulated; Fig. 1F). Across all four cell types, the proportion of induced and repressed transcripts was broadly balanced (Fig. 1G, H), suggesting coordinated activation and suppression of each cell type rather than a unidirectional transcriptional drift.

To distinguish the shared and cell type-specific responses, we performed Hallmark GSEA and summarized NES for significantly enriched pathways (*P*-adj <0.05) in each cell type (Fig. 1I). MCD elicited a conserved inflammatory signaling pathway across all four cell types, including interferon alpha and interferon gamma responses, TNF signaling via NF-κB, inflammatory response, and IL6-JAK-STAT3 signaling (Fig. 1I). In contrast, MCD uniformly induced a transcriptional suppression of core metabolic and bioenergetic processes, including cholesterol homeostasis, oxidative phosphorylation, and glycolysis, and cell cycle-associated pathways, such as E2F targets, G2M checkpoint, and MTORC1 signaling (Fig. 1I). This coordinated repression of metabolic and cell cycle pathways is consistent with a nutrient stress-induced metabolic restraint and proliferation pause described in models of hepatic injury and fibrogenesis. Collectively, these data demonstrate that MCD orchestrates transcriptional reprogramming across hepatocytes, macrophages, ECs, and stellate cells. Despite clear type-specific magnitude differences, a conserved inflammatory activation coupled with metabolic and cell cycle program suppression emerges as a unifying cross-cell type response to MCD.

### MCD stress drives hepatocyte detoxification and redox adaptation while suppressing sterol and lipid anabolism

**Fig. 2:**
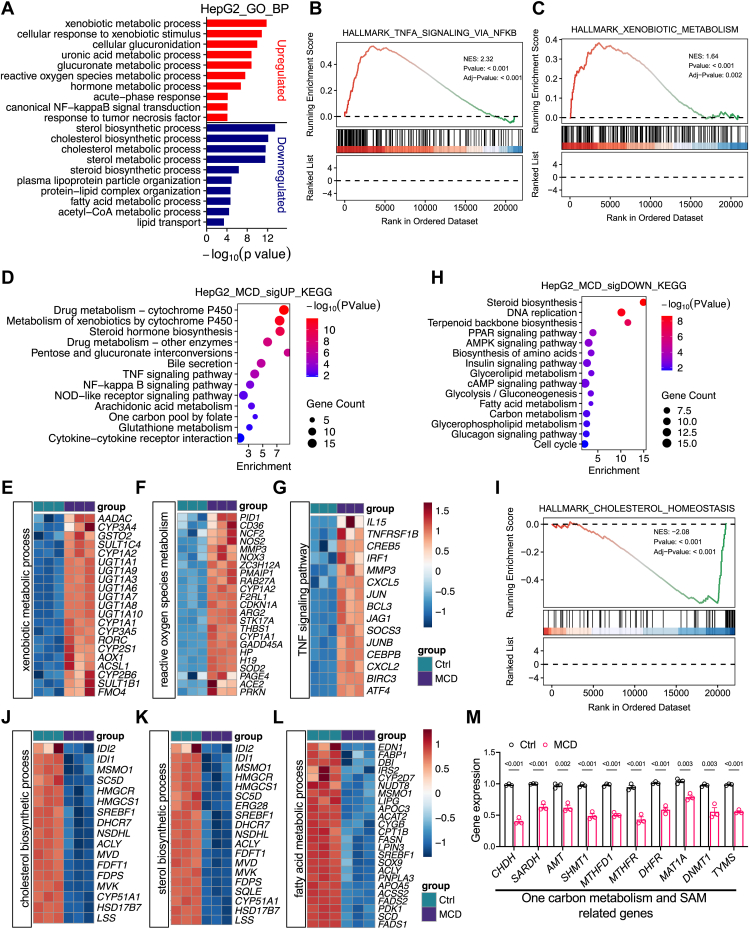
MCD stress drives hepatocyte detoxification and redox adaptation while suppressing lipid anabolism. A: GO biological process analysis of genes upregulated or downregulated by MCD medium in HepG2 cells. B and C: Hallmark GSEA of genes upregulated by MCD treatment in HepG2 cells. D: KEGG pathway enrichment analysis of genes induced by MCD treatment in HepG2 cells. E–G, Heatmap of genes related to xenobiotic metabolic process (E), reactive oxygen species metabolism (F), and TNF signaling pathway (G). H: KEGG pathway enrichment analysis of genes repressed by MCD treatment in HepG2 cells. I: Hallmark GSEA of genes negatively regulated by MCD treatment in HepG2 cells. J–L: Heatmaps of genes associated with cholesterol biosynthetic process (J), sterol biosynthetic process (K), and fatty acid metabolic process (L). M: Expression of genes related to one-carbon and SAM metabolism in HepG2 cells.

To understand MCD-induced remodeling of each cell type, we further conducted in-depth analysis on its cell type-specific impacts. HepG2 cells underwent the most extensive transcriptional reprogramming under MCD stress (Fig. 1C, G). GO enrichment analysis of upregulated genes revealed a prominent induction of hepatocyte detoxification and oxidative stress response pathway, including xenobiotic metabolic processes, cellular response to xenobiotic stimulus, glucuronidation, uranic acid metabolic process, and reactive oxygen species metabolic process (Fig. 2A). In parallel, stress response and inflammatory pathways, including canonical NF-κB signal transduction, response to TNF, and acute-phase response, were significantly enriched (Fig. 2A), indicating coordinated activation of hepatocyte defense and inflammatory programs under MCD stress. Hallmark GSEA further reinforced this induction of cell defense and inflammation by MCD. TNF signaling via NF-κB and xenobiotic metabolism were robustly enriched in MCD medium-treated HepG2 cells (Fig. 2B,C). MCD treatment was also associated with activation of apoptosis-related pathways and suppression of proliferative programs, including E2F targets and G2M checkpoint (supplemental Fig. S2A–D). KEGG pathway analysis further identified enrichment of drug metabolism cytochrome P450, metabolism of xenobiotics, pentose, and glucuronate interconversions, bile secretion, as well as TNF and NF-κB signaling (Fig. 2D).

Consistent with pathway analysis, heatmaps showed significant upregulation of core xenobiotic metabolism machinery, including multiple cytochrome P450 enzymes (*CYP3A4*, *CYP2B6*, and *CYP3A5*) and *UGT1A* family members (Fig. 2E). MCD also induced genes involved in oxidative stress adaption, including *NCF2*, *NOX3*, *NOS2*, and *SOD2* (Fig. 2F). The expression of genes involved in the activation and transduction of TNF signaling, including *IL15*, *TNFRSF1B*, *IRF1*, *CXCL2*, *CXCL5*, *JUN*, and *SOCS3*, was also increased (Fig. 2G), along with an induction of NF-κB signaling genes (supplemental Fig. S2E).

In contrast, genes downregulated by MCD were predominantly enriched for lipid and sterol anabolic processes. GO terms included cholesterol and sterol biosynthetic processes, plasma lipoprotein particle organization, fatty acid metabolic process, and lipid transport (Fig. 2A). KEGG analysis similarly highlighted steroid biosynthesis, fatty acid metabolism, glycerophospholipid metabolism, carbon metabolism, and cell cycle-related pathways (Fig. 2H). Hallmark cholesterol homeostasis was strongly enriched in downregulated genes (Fig. 2I). These suppressive effects were reflected by a significant downregulation of cholesterol synthesis and sterol biosynthetic genes, such as *HMGCR*, *HMGCS1*, *LSS*, and *SQLE* (Fig. 2J, K), together with a repression of fatty acid metabolism regulators, including *CPT1B*, *FASN*, *FABP1*, *ACSS2*, and *SCD* (Fig. 2L). These data indicate broad suppression of hepatocyte lipid anabolic capacity under MCD conditions.

Given that methionine and choline availability directly influences methyl group availability (47, 48, 49, 50, 51), we next interrogated the expression of SAM and one-carbon metabolism genes. MCD induced a compensatory transcriptional program characterized by activation of betaine-dependent demethylation and SAM cycle maintenance genes (*BHMT*, *DMGDH*, and *MAT2A*), upregulation of mitochondrial one-carbon metabolism components (*MTHFD2*, *MTHFD2L*, *MTHFD1L*, and *ALDH1L2*), and stimulation of trans-sulfuration in glutathione metabolism-related genes (*CTH*, *GCLC*, *GCLM*, and *TST*) (supplemental Fig. S2F, G). In contrast, core cytosolic folate-dependent one-carbon transfer and thymidylate synthesis genes were coordinately repressed, including *CHDH*, *SARDH*, *AMT*, *SHMT1*, *MTHFD1*, *MTHFR*, *DHFR*, and *TYMS* (Fig. 2M). Methionine adenosyltransferase *MAT1A* and SAM-dependent DNA methyltransferase *DNMT1* were also downregulated (Fig. 2M). This is consistent with diminished SAM synthesis and SAM-dependent methylation capacity. Collectively, these data indicate that MCD drives HepG2 cells into a cell defense-oriented state characterized by xenobiotic detoxification, redox adaptation, and inflammatory activation, while broadly suppressing sterol and lipid anabolic programs. MCD also remodels SAM metabolism by inducing compensatory mitochondrial one-carbon metabolism and trans-sulfuration, yet repressing cytosolic one-carbon metabolism and thymidylate synthesis with reduced SAM synthesis and methylation capacity.

### MCD-induced hepatocyte transcriptional programs are enriched in human MASH

**Fig. 3:**
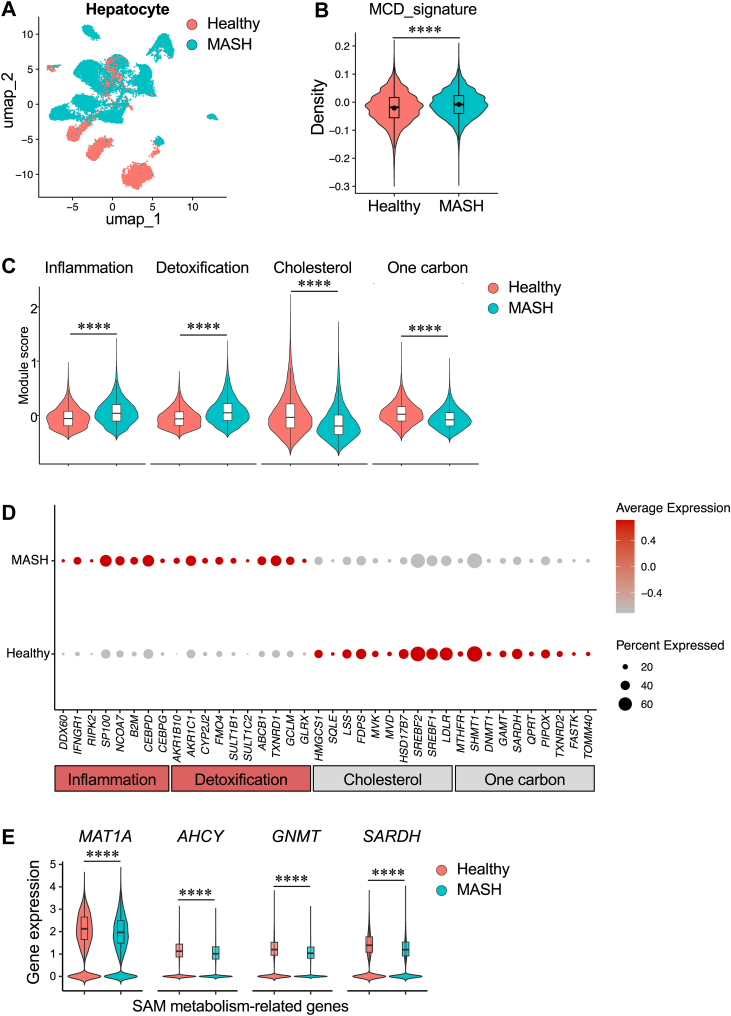
HepG2 MCD signatures are enriched in disease-associated hepatocyte states in human MASH. A: UMAP visualization of hepatocyte nuclei from a public human liver snRNA-Seq dataset (GSE244832) comparing healthy controls (N = 5) and MASH (N = 9). B: Module score distribution of the HepG2-derived MCD transcriptional signature across hepatocyte nuclei. C: Module scoring of key functional axes in hepatocytes, showing increased inflammation and detoxification programs and decreased cholesterol and one-carbon-related modules in MASH. D: Dot plot of representative transcripts illustrating concordant induction of inflammatory and xenobiotic metabolism genes and repression of cholesterol biosynthetic genes in MASH hepatocytes. Dot size indicates the fraction of nuclei expressing the indicated genes, and color intensity denotes average expression level. E: Violin plots of methionine cycle and SAM pathway transcripts (including *MAT1A*, *AHCY*, *GNMT*, and *SARDH*) in hepatocyte nuclei from healthy and MASH livers.

To determine whether the transcriptional programs induced by MCD treatment in vitro are relevant to human MASH, we projected the MCD-responsive gene signature derived from HepG2 cells onto a human liver snRNA-Seq dataset generated from clinically and histologically characterized donor livers (GSE244832) (52). In this cohort, deidentified livers were classified by a liver pathologist according to MASH Clinical Research Network (CRN) criteria: normal livers (CRN score <3; N = 5) and MASH livers (CRN score ≥5; N = 9, fibrosis stage 2–4), with diagnoses defined by combined histological evaluation and clinical history. UMAP visualization of hepatocytes revealed clear disease-associated transcriptional remodeling, with distinct separation between normal and MASH samples (Fig. 3A). Module scoring demonstrated significant enrichment of the MCD-induced gene signature in hepatocytes from MASH livers relative to normal controls (Fig. 3B), indicating that the transcriptional program elicited by methyl donor deficiency in vitro is activated in human MASH.

We next interrogated whether the major functional axes identified in MCD-treated HepG2 cells were similarly altered in vivo in human MASH. In hepatocytes from MASH livers, module scores for inflammatory signaling and detoxification/xenobiotic metabolism were significantly elevated, whereas cholesterol biosynthesis and one-carbon/SAM metabolism modules were suppressed (Fig. 3C). These changes parallel the transcriptional reprogramming observed in MCD-treated HepG2 cells. Gene expression analyses further supported this convergence. Representative inflammatory and xenobiotic metabolism genes exhibited increased expression in human MASH hepatocytes, whereas multiple cholesterol biosynthetic enzymes were reduced (Fig. 3D). Importantly, key components of SAM and one-carbon metabolism, including *MAT1A*, *AHCY*, *GNMT*, and *SARDH*, were significantly downregulated in human MASH hepatocytes compared with healthy subjects (Fig. 3E). Together, these data indicate that MCD-induced hepatocyte transcriptional reprogramming in vitro converges with disease-associated hepatocyte states in human MASH and represents a mechanistically relevant component of hepatocyte dysfunction in human MASH.

### ECs exhibit inflammatory activation and lipid metabolism repression under MCD stress

**Fig. 4:**
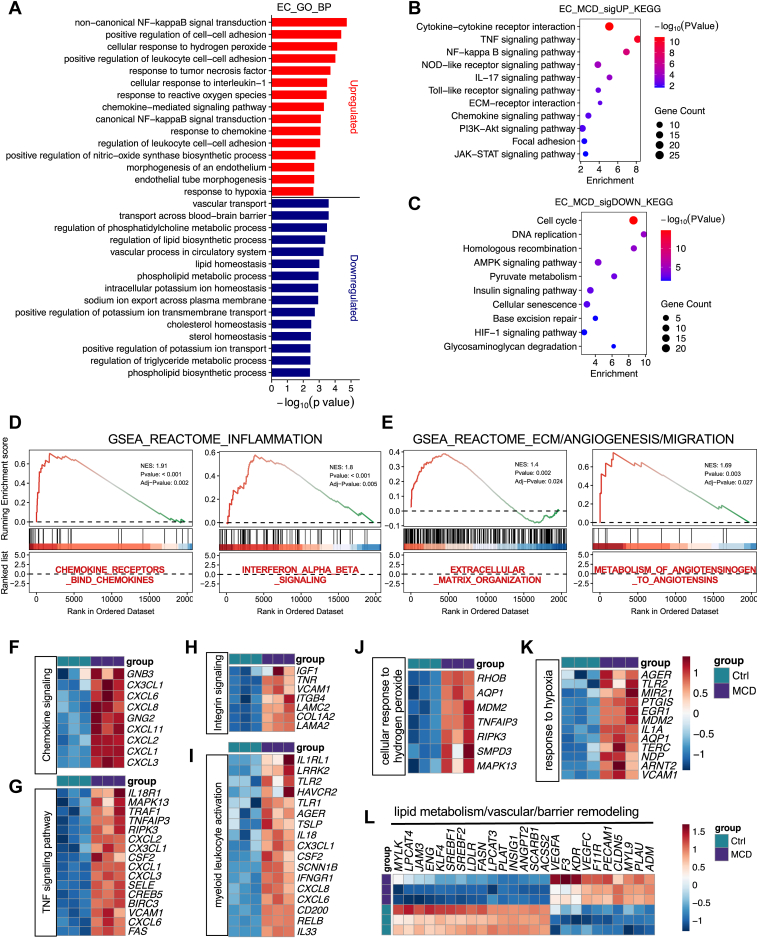
ECs exhibit inflammatory activation and lipid metabolism repression under MCD stress. A: GO biological process enrichment of DEGs in ECs after MCD treatment. Top enriched terms for upregulated genes are shown in red and downregulated genes are shown in blue. B and C: KEGG pathway enrichment of upregulated (B) and downregulated (C) genes in ECs under MCD conditions. D and E: Representative Reactome GSEA plots summarizing genes regulated by MCD treatment in ECs. F–L: Heatmaps of endothelial genes related to chemokine signaling (F), TNF signaling (G), integrin signaling (H), myeloid leukocyte activation (I), cellular response to hydrogen peroxide (J), hypoxia-associated response (K), and lipid homeostasis and vascular barrier remodeling (L) in MCD-treated ECs relative to controls. DEG, differently expressed gene.

Liver sinusoidal ECs become dysfunctional and undergo capillarization during MASH, contributing to impaired lipid exchange, hypoxia, and amplification of inflammatory and fibrogenic signaling (53, 54, 55). They mediate monocyte recruitment through upregulation of adhesion molecules and chemokines, thereby amplifying inflammation and fibrogenic signaling in MASH (56, 57, 58). GO enrichment revealed a coordinated bidirectional remodeling of endothelial gene expression (Fig. 4A). Terms enriched among MCD-upregulated genes included canonical and noncanonical NF-κB signaling, responses to TNF and IL-1, chemokine-mediated signaling, regulation of leukocyte adhesion, and cellular responses to reactive oxygen species and hypoxia. In parallel, negatively enriched terms included lipid and phospholipid processes, indicating repression of lipid homeostatic and barrier-maintenance functions. KEGG pathway analysis supported this coordinated shift (Fig. 4B, C). Inflammatory and innate immune pathways, including cytokine-cytokine receptor interaction, TNF and NF-κB signaling, NOD-like and Toll-like receptor pathways, IL-17 signaling, JAK-STAT signaling, and ECM organization, were significantly enriched. Pathways associated with cell cycle, DNA replication, AMP-activated protein kinase (AMPK) signaling, pyruvate metabolism, insulin signaling, and hypoxia-inducible factor 1 signaling were reduced, consistent with metabolic and proliferative restraint. Reactome GSEA further reinforced enrichment of inflammatory and ECM-related programs, including chemokine receptor signaling, interferon α/β signaling, ECM organization, and metabolism of angiotensinogen to angiotensin (Fig. 4D, E and supplemental Fig. S3), as well as suppression of mitotic cell cycle, cholesterol biosynthesis, RHO GTPase signaling, and respiratory electron transport. MCD-induced chemokine and adhesion molecules (*CX3CL1*, *CXCL1/2/3/6/8*, and *VCAM1*), NF-κB-associated mediators (*TNFAIP3*, *TRAF1*, *BIRC3*, and *RELB*), together with innate immune sensors (*TLR1*, *TLR2*, and *AGER*) (Fig. 4F–I), supporting enhanced immune cell recruitment and endothelial-immune crosstalk. Oxidative stress and hypoxia-responsive genes were also elevated (Fig. 4J, K), supporting a vascular stress state.

In contrast, downregulated gene sets were enriched for lipid homeostasis and endothelial metabolic regulation. GO and KEGG analyses identified suppression of phospholipid metabolism, cholesterol and sterol homeostasis, triglyceride metabolism, AMPK signaling, insulin signaling, and hypoxia-inducible factor 1 signaling. Cholesterol and lipid homeostasis, including *SREBF1*, *SREBF2*, *LDLR*, *FASN*, *INSIG1*, *SCARB1*, and *ACSS2*, as well as phospholipid remodeling enzymes *LPCAT3* and *LPCAT4* (Fig. 4L). The endothelial homeostasis-associated transcription factor *KLF4* also declined. This is consistent with a disruption of homeostatic endothelial function (Fig. 4L). Importantly, MCD elicited an EC-specific upregulation of pathways marked by the VEGF-VEGF receptor axis (*VEGFA*, *VEGFC*, and *KDR*) and the procoagulant and proteolytic genes, *F3* and *PLAU* (Fig. 4L). Cell junction and cytoskeleton regulation were also rewired, as shown by the upregulation of *F11R*, *PECAM1*, *CLDN5*, *MYL9*, and *ADM* (Fig. 4L). The enhanced VEGF signals and vascular remodeling were reported as major factors mediating EC capillarization and altering hepatic vessel permeability in MASH (59, 60). Overall, these MCD-driven repressions of lipid and phospholipid metabolism and activations of vascular inflammation and remodeling could contribute to pathological changes of EC functions in MASH.

**Fig. 5:**
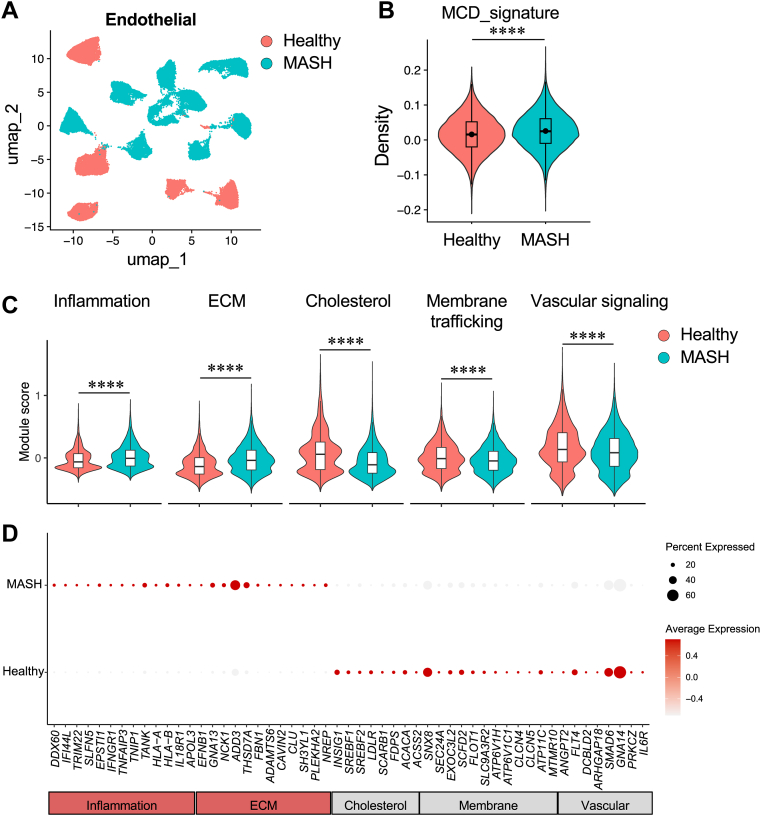
Human MASH ECs exhibit enrichment of the MCD transcriptional signature. A: UMAP visualization of human hepatic ECs from healthy controls and MASH livers (GSE244832), colored by disease status. B: Violin plot showing MCD-induced endothelial signature scores in human ECs from healthy and MASH samples. The signature was generated from the top 100 upregulated and top 100 downregulated genes in MCD-treated EC bulk RNA-Seq and projected onto the human snRNA-Seq dataset for module scoring. C: Violin plots of module scores for inflammation, ECM, cholesterol metabolism, membrane trafficking, and vascular signaling programs in ECs from healthy and MASH livers. D: Dot plot displaying representative genes grouped by functional category (inflammation, ECM remodeling, cholesterol metabolism, membrane trafficking, and vascular signaling). Dot size indicates the fraction of cells expressing the gene, and color intensity represents the average expression level.

We next examined whether the endothelial transcriptional program induced by MCD recapitulates the hepatic EC transcriptome in human MASH. We projected the MCD-induced endothelial gene signature onto human endothelial snRNA-Seq from the same dataset (GSE244832). UMAP visualization of ECs demonstrated disease-associated remodeling of endothelial transcriptome in MASH compared with healthy subjects (Fig. 5A). Module scoring revealed significant enrichment of the MCD signature in ECs from MASH livers (Fig. 5B). Consistent with the in vitro findings, ECs in MASH exhibited increased inflammatory and ECM remodeling modules and reduced cholesterol metabolism, membrane trafficking, and vascular signaling programs (Fig. 5C). Human MASH ECs showed higher expression of interferon and inflammatory response genes, including *DDX60*, *IFI44L*, *TRIM22*, *SLFN5*, *EPSTI1*, *TNFAIP3*, *TNIP1*, *TANK*, and *IFNGR1*, and ECM remodeling genes, such as *THSD7A*, *ADAMTS6*, and *CLU*. In contrast, healthy ECs preferentially expressed lipid regulatory genes, including *INSIG1*, *SREBF1*, *SREBF2*, *LDLR*, *SCARB1*, *FDPS*, *ACACA*, and *ACSS2*, as well as vascular signaling and membrane trafficking genes, including *ANGPT2*, *FLT4*, *SMAD6*, and *ATP11C* (Fig. 5D). Together, these data demonstrated that MCD induces an endothelial transcriptional shift characterized by inflammation, ECM remodeling, and repression of lipid homeostasis, which closely parallels endothelial dysfunction observed in human MASH.

### MCD activates inflammatory and phagocytic pathways while repressing lipid and one-carbon metabolism in macrophages

**Fig. 6:**
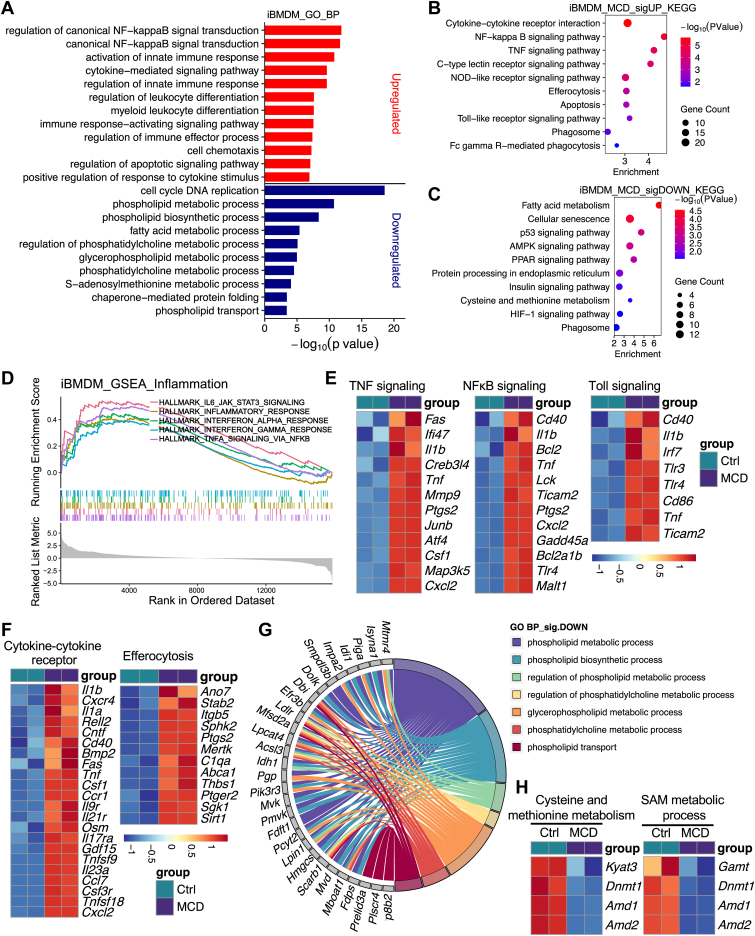
MCD activates inflammatory and phagocytic pathways while repressing lipid metabolism in macrophages. A: GO biological process enrichment of DEGs in iBMDM following MCD treatment. B and C: KEGG pathway enrichment of upregulated (B) and downregulated (C) genes in iBMDM under MCD conditions. D: Representative GSEA plots summarizing genes regulated by MCD treatment in iBMDM. E and F: Heatmaps of representative macrophage gene expression in iBMDM. G: Chord diagram summarizing MCD-repressed lipid and phospholipid metabolism genes in iBMDMs. H: Heatmap of genes related to cysteine and methionine metabolism and the SAM metabolic process in MCD-treated iBMDMs. DEG, differently expressed gene.

Monocyte-derived macrophages play critical roles in the pathogenesis of MASH, as they mediate hepatic inflammation, induce hepatocyte injury, and promote fibrosis (24, 61, 62, 63). In macrophages treated with MCD medium, upregulated genes were enriched in inflammatory pathways, including canonical NF-κB signaling, cytokine-mediated signaling, regulation of leukocyte differentiation, chemotaxis, and immune effector processes (Fig. 6A). KEGG analysis of upregulated genes highlighted cytokine-to-cytokine receptor interaction, NF-κB, and TNF signaling, Toll-like and NOD-like receptor pathways, along with phagosome and Fc gamma receptor-mediated phagocytosis pathways (Fig. 6B). In contrast, downregulated genes were clustered in lipid metabolism and regulation processes, including phospholipid and glycerophospholipid metabolism, phosphatidylcholine metabolism, fatty acid metabolism, and AMPK and PPAR signaling (Fig. 6A, C). GSEA further validated this shift of transcriptional profile in MCD-treated iBMDM, showing that upregulated genes are enriched in hallmark inflammatory responses, TNF signaling via NF-κB, IL6-JAK-STAT3 signaling, and interferon α,and interferon γ responses (Fig. 6D).

MCD markedly induced proinflammatory mediators (*Il1b*, *Tnf*, *Ptgs2*, and *Cd40*), immediate early response genes (*Junb*, *Atf4*), and upstream Toll-like signaling components (*Tlr3*, *Tlr4*, *Irf7*, and *Ticam2*) (Fig. 6E). In parallel, macrophage effector functions were enhanced, as reflected by elevated expression of efferocytosis and phagocytic genes, including *Mertk*, *C1qa*, *Abca1*, *Thbs1*, *Stab2*, and *Itgb5*, as well as induction of cytokine and chemokine signaling genes (*Il1a*, *Il1b*, *Cxcr4*, *Relb*, *Csf1*, *Ccr1*, and *Osm*) (Fig. 6F). Concomitant with inflammatory activation, MCD results in a significant downregulation of genes involved in phospholipid metabolism (Fig. 6G), including genes of phosphatidylcholine metabolism (*Mfsd2a*, *Lpcat4*, *Scarb1*, *Ldlr*, and *Dbi*), phospholipid transport (*Mfsd2a*, *Atp8b2*, *Ldlr*, *Scarb1*, and *Plscr4*), phospholipid biosynthesis and glycerophospholipid metabolism (*Pik3r3*, *Mboat1*, *Pcyt2*, *Lpin1*, *Msfd2a*, *Acsl3*, and related nodes), as well as regulators of phosphatidylcholine metabolic processes (*Mfsd2a*, *Scarb1*, *Acsl3*, and *Ldlr*) (supplemental Fig. S4A–E). Notably, MCD also downregulated genes involved in cysteine and methionine metabolism and the SAM metabolic process (*Gamt*, *Dnmt1*, *Amd1*, and *Amd2*), suggesting disruption of one-carbon metabolism in macrophages (Fig. 6H). Together, these data indicate that MCD drives macrophages toward an inflammatory and phagocytic state while coordinately repressing lipid and one-carbon metabolism.

**Fig. 7:**
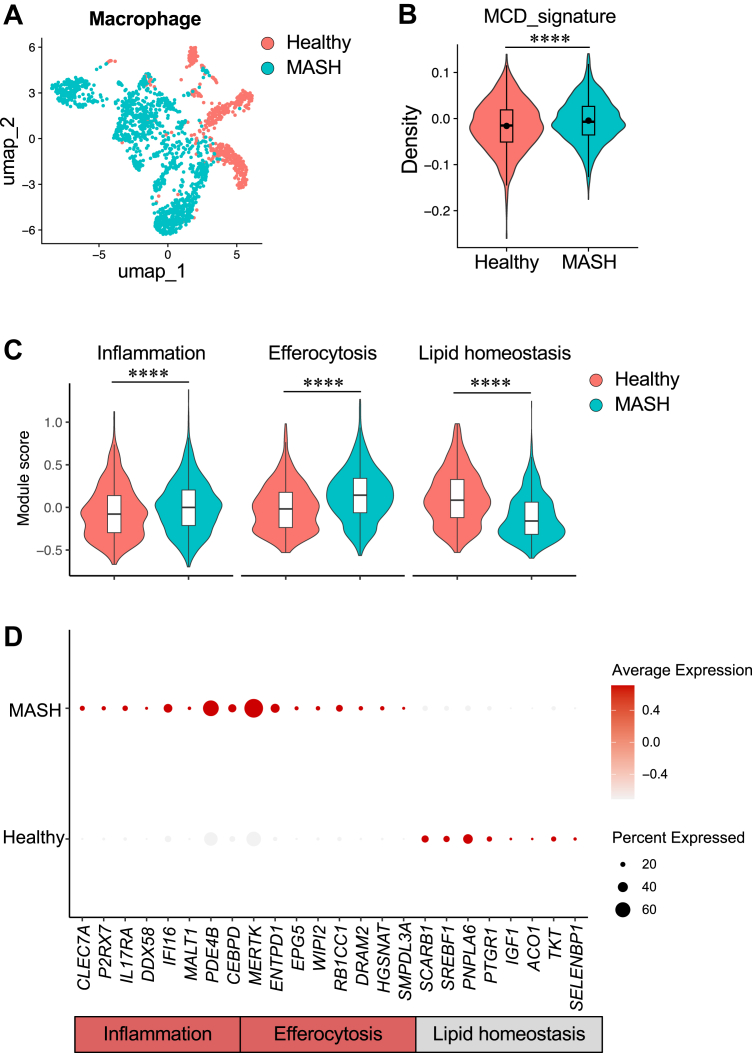
MCD-induced macrophage transcriptional signatures map to disease-associated macrophage states in human MASH. A: UMAP of human liver macrophages colored by disease status (healthy vs. MASH). B: Violin plot showing MCD-induced macrophage signature scores in human liver macrophages from healthy and MASH samples. The signature was derived from the top 100 upregulated and top 100 downregulated genes identified by bulk RNA-Seq of MCD-treated iBMDMs and projected onto the human snRNA-Seq dataset for module scoring. C: Violin plots of module scores in human macrophages summarizing inflammation, efferocytosis, and lipid homeostasis programs from healthy and MASH livers. D: Dot plot showing average expression (color intensity) and percentage of expressing cells (dot size) for representative genes grouped by inflammation, efferocytosis, and lipid homeostasis programs in healthy and MASH macrophages.

To assess in vivo relevance, we projected the MCD-induced macrophage signature onto human liver macrophage snRNA-Seq from the same dataset (GSE244832). Consistent with previous reports, MASH substantially reprogrammed the transcriptome of liver macrophages (Fig. 7A). The MCD signature score was significantly higher in macrophages from MASH livers than in healthy livers (Fig. 7B), indicating enrichment of the MCD-associated program in human disease. Module scoring further showed increased expression of inflammation and efferocytosis-related genes in human MASH macrophages, accompanied by reduced lipid metabolism genes (Fig. 7C). MASH-associated liver macrophages showed higher expression of inflammatory and innate immune mediators, including *CLEC7A*, *P2RX7*, and *IL1B*, as well as interferon-associated genes, such as *DDX58*, *IFI16*, and *MX1* (Fig. 7D). In contrast, macrophages from healthy livers exhibited higher expression of lipid metabolism and hemostatic genes, including *SCARB1*, *PNPLA6*, *IGF1*, *ACO1*, and *TKT* (Fig. 7D). Together, these data show that MCD-induced inflammatory activation, while suppressing lipid metabolism and SAM metabolism in macrophages, closely parallels transcriptional features of liver macrophages in human MASH.

### MCD triggers the transition of HSCs from a lipid-storing quiescent state to a profibrotic phenotype

**Fig. 8:**
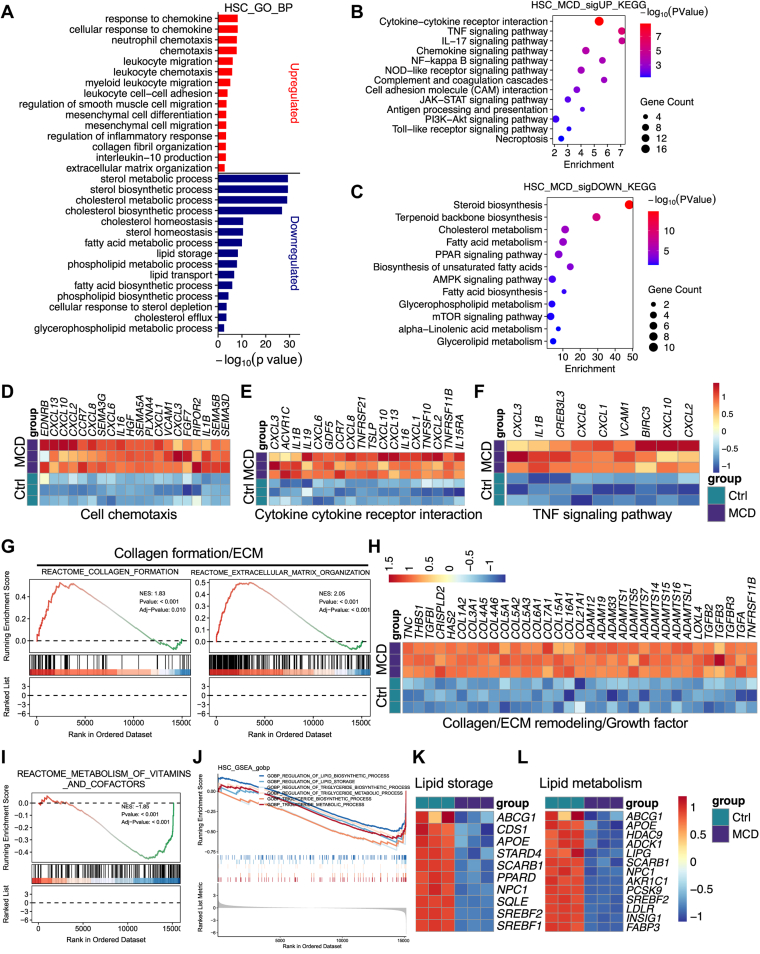
MCD induces a profibrotic transcriptional program in HSCs characterized by ECM remodeling and repression of lipid and vitamin metabolic pathways. A: GO biological process enrichment analysis of upregulated (red) or downregulated (blue) genes by MCD medium in human HSCs. B and C: KEGG pathway enrichment analysis of upregulated (B) and downregulated (C) genes by MCD medium in human HSCs. D–F: Heatmaps of genes involved in chemotaxis (D), cytokine-cytokine receptor interaction (E), and TNF signaling (F) in human HSCs. G: GSEA enrichment plot for reactome collagen formation and ECM organization induced by MCD medium in human HSCs. H: Heatmap of ECM, collagen remodeling, and growth factor-related genes induced by MCD medium in human HSCs. I: Reactome GSEA enrichment plots showing negative regulation of the vitamin-associated pathway in MCD-treated HSCs. J: GSEA enrichment plots showing pathways negatively regulated by MCD in human HSCs. K and L: Heatmaps of genes related to lipid storage (K) and lipid homeostasis (L).

HSCs are the principal effector cells of liver fibrosis and undergo activation during MASH, transitioning from a quiescent, lipid-storing state to a myofibroblast-like phenotype that produces ECM (64, 65, 66). To define how MCD reshapes MASH-associated fibrogenesis, we next profiled the transcriptome of HSCs cultured in control or MCD medium. GO biological process analysis showed that MCD-upregulated transcripts were significantly enriched in inflammatory and chemokine response pathways, including responses to chemokines and chemokine-mediated signaling, neutrophil chemotaxis and leukocyte migration, regulation of inflammatory response, and mesenchymal cell migration (Fig. 8A). Processes linked to ECM organization, collagen fibril organization, and smooth muscle-associated differentiation were also significantly enriched, indicating a profibrotic phenotype (Fig. 8A). In contrast, downregulated genes were significantly enriched for sterol and cholesterol biosynthetic and metabolic processes, cholesterol homeostasis, fatty acid metabolism, lipid storage, and lipid transport, as well as phospholipid and glycerophospholipid metabolic and biosynthetic pathways. Consistently, KEGG analysis of upregulated genes highlighted activation of cytokine-cytokine receptor interaction and major inflammatory signaling pathways, including TNF, NF-κB, IL-17, chemokine, and JAK-STAT signaling (Fig. 8B). Hallmark GSEA similarly supported enrichment of inflammatory programs in MCD-treated HSCs (supplemental Fig. S5A). Conversely, downregulated genes showed suppression of steroid biosynthesis, cholesterol metabolism, fatty acid metabolism, and glycerophospholipid metabolism (Fig. 8C).

MCD-induced inflammatory activation of HSC resulted in a significantly upregulated expression of chemokine and cytokine genes, including *CXCL2*, *CXCL3*, *CXCL6*, *CXCL8*, *IL1B*, and downstream signaling mediator genes, suggesting that activated HSC contributes to the chemotaxis of immune cells, thus further promoting inflammation in a vicious cycle (Fig. 8D–F and supplemental Fig. S5B). Beyond inflammatory activation, MCD also activates ECM remodeling pathways that mediate hepatic fibrosis. GSEA revealed an enrichment of collagen-centered and fibrogenesis-associated pathways, including collagen formation, ECM organization, crosslinking of collagen fibrils, and integrin cell surface interactions, within the transcriptionally upregulated gene set in MCD medium-treated HSCs (Fig. 8G and supplemental Fig. S5C). Heatmap showed a significant upregulation of canonical ECM and remodeling genes, including *COL1A2*, *COL3A1*, *COL5A1*, *COL5A2*, *COL6A1*, multiple *ADAMTS* family members, and profibrotic growth factor signaling components, such as *TGFB2* and *TGFB3* (Fig. 8H). These data indicate that MCD stress induces both inflammatory and chemotactic signaling and fibrogenic processes in human HSCs.

In contrast to the induction of inflammatory and fibrogenic processes, MCD elicited a broad suppression of metabolic processes in human HSCs. Reactome GSEA revealed this metabolic suppression, revealing an enrichment of cholesterol biosynthesis and cholesterol metabolism (Fig. S5D), vitamin transport and metabolism (Fig. 8I and supplemental Fig. S5E), and multiple lipid anabolic and storage pathways, including triglyceride biosynthesis and metabolism, lipid biosynthesis and storage, phospholipid metabolism and biosynthesis, and fatty acid metabolic programs (Fig. 8J and supplemental Fig. S5F, G). This repression of lipid metabolism pathways aligns with a core hallmark of HSC activation, in which HSCs lose lipid droplet and retinoid and enhance cytoskeletal remodeling, migratory capacity, and ECM production. Expression of genes involved in lipid metabolism and storage, such as *ABCG1*, *APOE*, *NPC1*, *SQLE*, *SREBF1*, and *SREBF2*, was significantly reduced (Fig. 8K, L). Together, these findings indicate that MCD stress could drive a marked transition of HSC from a quiescent state featured by lipid metabolism and storage function to an activated state characterized by its proinflammatory and fibrogenic function.

**Fig. 9:**
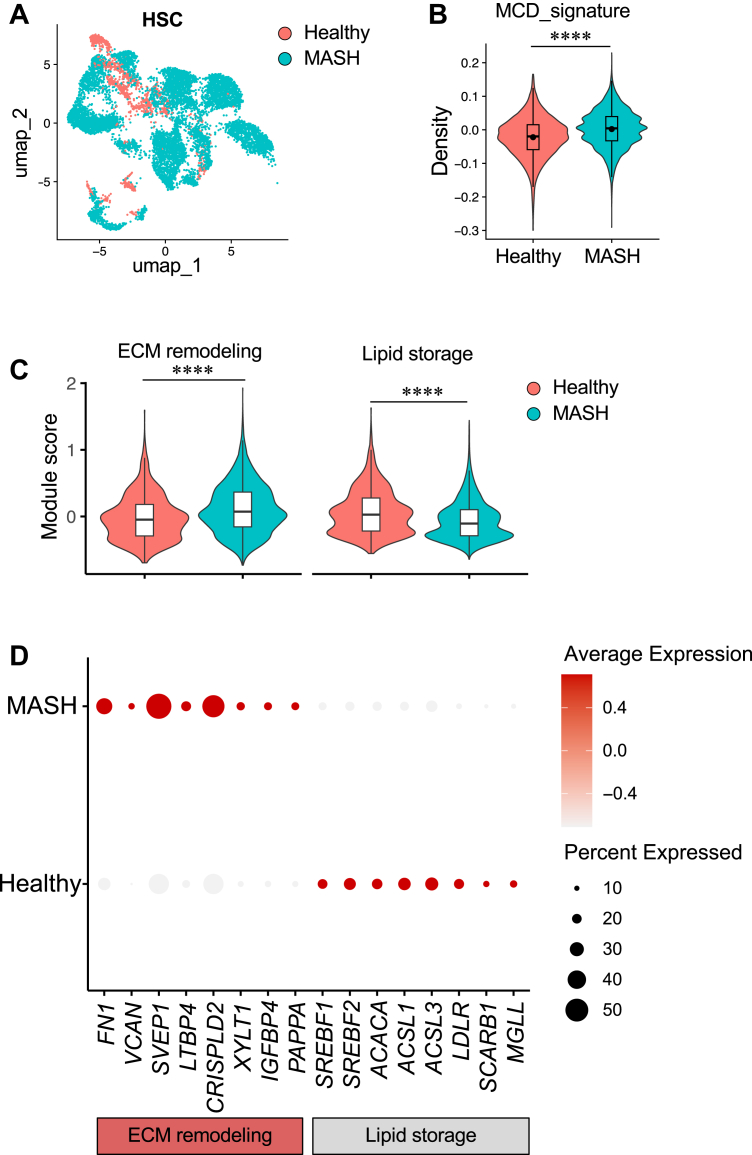
Enrichment of the MCD-derived transcriptional signature in human MASH HSCs. A: UMAP visualization of HSC nuclei from the human liver snRNA-Seq dataset (GSE244832) comparing healthy controls and MASH samples. B: Module score distribution of the HSC-derived MCD transcriptional signature across human MASH HSC nuclei, demonstrating increased signature scores in MASH relative to healthy controls. C: Violin plots of module scores in HSC nuclei highlighting increased ECM remodeling programs and reduced lipid storage-associated pathways in MASH. D: Dot plot of representative genes illustrating induction of ECM remodeling genes (*FN1*, *VCAN*, *SVEP1*, *LTBP4*, *CRISPLD2*, *XYLT1*, and *IGFBP4*) and repression of lipid storage and sterol regulatory genes (*SREBF1*, *SREBF2*, *ACACA*, *ACSL1*, *ACSL3*, *LDLR*, *SCARB1*, and *MGLL*) in human MASH HSCs. Dot size indicates the fraction of expressing nuclei, and color denotes the average expression level.

Analysis of human HSC snRNA-Seq from the same dataset (GSE244832) demonstrated substantial transcriptional remodeling of HSC in MASH (Fig. 9A). Projection of the MCD-induced HSC signature revealed elevated signature scores in MASH HSCs compared with healthy controls (Fig. 9B). Consistent with the in vitro MCD signatures, human MASH HSCs exhibited increased ECM remodeling gene expression and reduced lipid storage-associated gene expression (Fig. 9C, D). Together, these results indicate that MCD-induced HSC transcriptional programs parallel key features of disease-associated HSC states in human MASH.

## Discussion

A major challenge in understanding the pathogenesis of MASH is to elucidate how nutrient deficiency stress causes the inflammatory and fibrogenic states across distinct cell types and govern clinical outcomes (11). As essential nutrients for human health, the lack of methionine and choline disrupts one-carbon metabolism and SAM synthesis. SAM deficiency promotes lipid accumulation, inflammation, and progression toward steatohepatitis and fibrosis (20, 21, 22, 23). In preclinical studies, multiple types of MCD diets have been widely used to establish the MASH mouse model. However, the cell-intrinsic impacts of MCD on distinct hepatic cell types and how the MCD diet causes MASH are still poorly understood. In this study, an amino acid-defined MCD medium was used to interrogate how methionine and choline depletion transcriptionally regulate functions of four different types of cells that contribute to MASH pathogenesis. The results showed that, across all four major cell types, MCD medium induced inflammatory and stress response pathways, while repressing metabolism and proliferation pathways. This pattern suggests that lack of methionine and choline is sufficient to impose a common proinflammatory effect while disrupting normal metabolic processes, particularly lipid metabolism, thus leading to the development of MASH.

Methionine and choline availability constrains the capacity to generate SAM, the universal methyl donor for methyl transfer reactions that influence chromatin regulation, RNA processing, protein modification, and lipid methylation (19). Choline availability also constrains phosphatidylcholine biosynthesis through the CDP choline pathway and impacts methyl donor flux through betaine-dependent remethylation reactions (18). Low dietary choline intake has also been associated with metabolic dysfunction-associated steatotic liver disease prevalence and fibrosis risk (67). Consistently, reduced hepatic SAM has been reported in chronic liver diseases (22, 68, 69). Both clinical and preclinical evidence supported the benefits of SAM in liver diseases, underscoring the need to further define how this methyl donor metabolism contributes to chronic liver diseases (20, 22, 70, 71). In this context, profiling the MCD-induced transcriptional changes across four liver cell types and comparing them with transcriptomic data from human MASH provide a valuable framework to understand how the disruption of one-carbon and phosphatidylcholine metabolism contributes to MASH pathogenesis.

Under MCD stress, hepatocytes showed the most robust transcriptional reprogramming, which is dominated by an induction of detoxification and oxidative stress response pathways, and a suppression of sterol and lipid anabolism. This combination is consistent with a nutrient stress response: defense and detoxification programs are amplified, whereas lipid synthesis pathways are curtailed. The simultaneous upregulation of one-carbon and glutathione metabolism genes likely indicates a coordinated stress adaptation that integrates redox buffering with altered methyl donor metabolism. These MCD-induced hepatocyte function shifts recapitulate disease-associated hepatocyte states in human MASH, where inflammatory and detoxification pathways are enhanced and cholesterol metabolism is reduced. This observation supports the concept that a biochemically defined nutrient stress can mimic components of disease-associated hepatocyte states, providing mechanistic insight that complements descriptive single-cell atlases.

In ECs, MCD-induced inflammatory response promotes endothelial activation processes characterized by increased chemokine signaling, adhesion and immune interaction factors, and matrix remodeling. MCD medium also results in a transcriptional repression of lipid and phospholipid metabolism and cell proliferation. These outputs align with established roles of hepatic ECs as immune response regulators whose dysfunction promotes immune cell recruitment, vascular remodeling, and fibrogenic progression. In advanced human fibrosis, single-cell studies have identified scar-associated ECs that promote leukocyte transmigration. We observed a similar endothelial activation in MCD-treated cells, which overlapped with the transcriptional profile observed in human MASH endothelium. Together, these findings suggest that nutrient stress could contribute to vascular remodeling, thereby exacerbating inflammation and promoting fibrogenesis.

Macrophages treated with MCD medium exhibited innate immune activation and phagocytic remodeling, along with suppression of phospholipid and SAM metabolism. The macrophage response to MCD recapitulated key features of disease-associated states of monocyte-derived macrophage in MASH, including inflammatory activation, chemokine induction, and remodeling of phagocytic programs (72). This observation provides a plausible mechanism by which SAM and phosphatidylcholine constraints could intensify macrophage inflammatory polarization while reshaping phagocytic programs that influence hepatocyte injury resolution and HSC activation signals. In HSCs, MCD triggered a robust transcriptional activation of chemotaxis and ECM remodeling and suppressed lipid and sterol metabolism pathways. This transcriptomic reprogramming of HSCs aligned with features of disease-associated HSC in human MASH, indicating a switch of HSC from quiescent state to activated state. The activated HSCs promote hepatic fibrosis while further exacerbating immune cell infiltration and inflammation in MASH.

In vitro cultured systems have limitations, including the lack of the complex tissue microenvironment and multicellular interactions present in vivo, and therefore cannot fully recapitulate the pathophysiology of the MASH liver. Therefore, we compared the in vitro transcriptional regulation with human snRNA-Seq data from MASH livers to validate the physiological relevance of the transcriptional programs identified in our cultured cell datasets. Human single-cell/nucleus studies of MASH and liver fibrosis have emphasized that fibrotic progression is organized within spatially restricted multicellular niches, in which scar-associated macrophages, activated ECs, and collagen-producing stromal cell populations coordinate immune recruitment, vascular remodeling, and matrix deposition (14). Our cross-cell type analysis supports this niche model from a metabolic perspective. Disruption of SAM and phosphatidylcholine metabolism by methionine and choline depletion triggers a common inflammatory response across cell types, while also inducing cell type-specific changes. ECs and macrophages promote immune cell recruitment, and HSCs activate chemotaxis and matrix remodeling pathways. Clinically, the coordinated nature of this response suggests that fibrotic progression is sustained by parallel changes in hepatocytes, ECs, macrophages, and HSCs, highlighting the need to simultaneously target multiple pathways that maintain HSC activation and ECM deposition. More broadly, linking defined nutrient stress in the MCD model to human single-nucleus transcriptomics data connects a controlled metabolic perturbation to disease-associated cellular states. These findings may inform future use of the MCD models to interrogate specific pathological features of MASH and may facilitate the development of targeted therapeutic strategies for MASH and liver fibrosis.

## Data availability

All data necessary to evaluate the conclusions of this study are included in the article and its supplemental data files or have been deposited in the GEO database under accession number GSE319635. Additional data supporting the findings of this study are available from the corresponding author upon request.

## Supplemental data

This article contains supplemental data.

## Conflict of interest

The authors declare that they have no conflicts of interest with the contents of this article.
