# Supplementary material for: Methionine and choline deficiency rewires transcriptional programs to recapitulate molecular features of human MASH
Source: J Lipid Res. 2026 Mar 18;67(4):101022. doi: 10.1016/j.jlr.2026.101022 (PMC13091525; doi:10.1016/j.jlr.2026.101022)
Supplement: Supplementary figures [file mmc1.pdf]

Figure S1

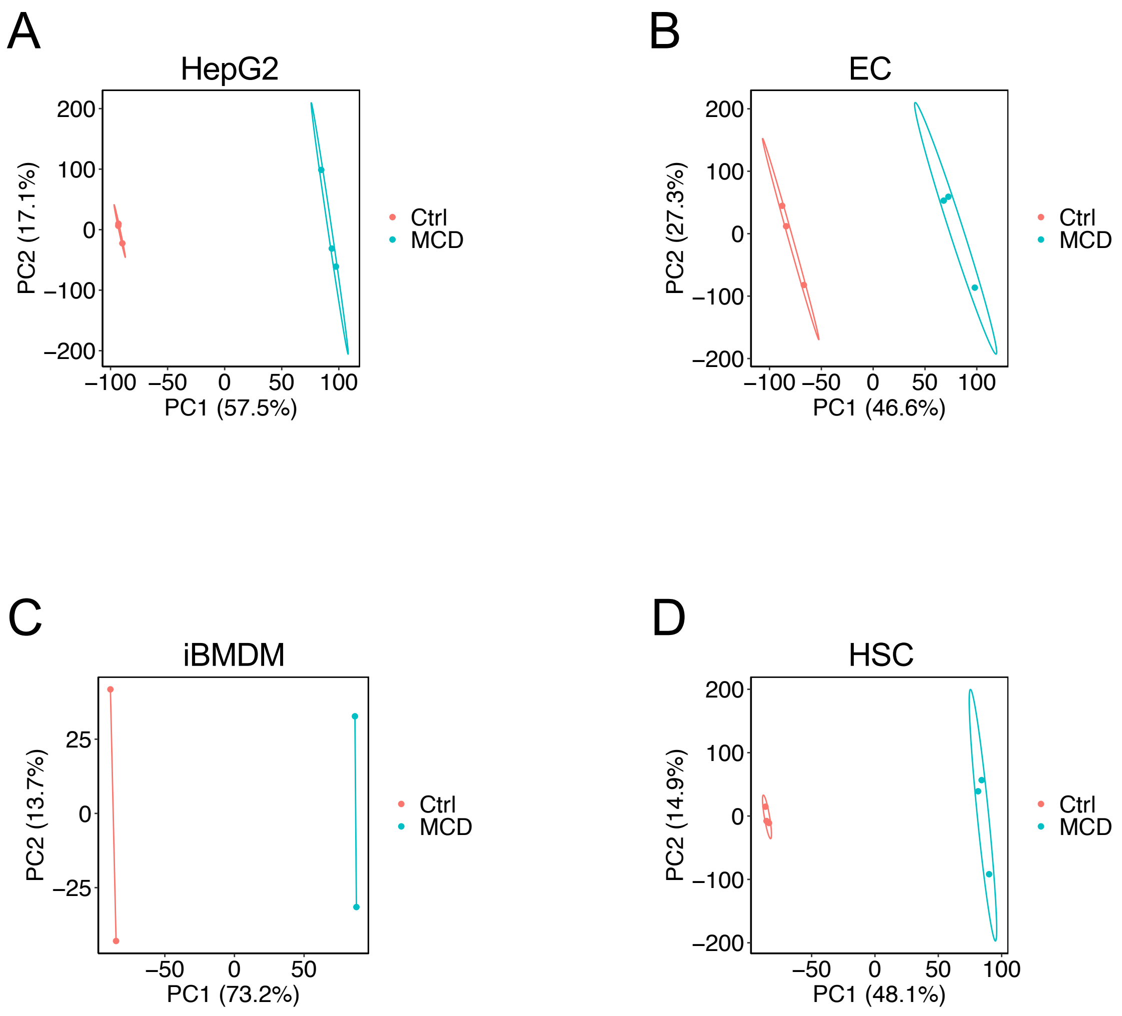
A
HepG2
PC2 (17.1%)
PC1 (57.5%)
200
100
0
−100
−200
−100
−50
0
50
100
Ctrl
MCD
B
EC
PC2 (27.3%)
PC1 (46.6%)
200
100
0
−100
−200
−100
−50
0
50
100
Ctrl
MCD
C
iBMDM
PC2 (13.7%)
PC1 (73.2%)
25
0
−25
−50
0
50
Ctrl
MCD
D
HSC
PC2 (14.9%)
PC1 (48.1%)
200
100
0
−100
−200
−50
0
50
100
Ctrl
MCD

Figure S2

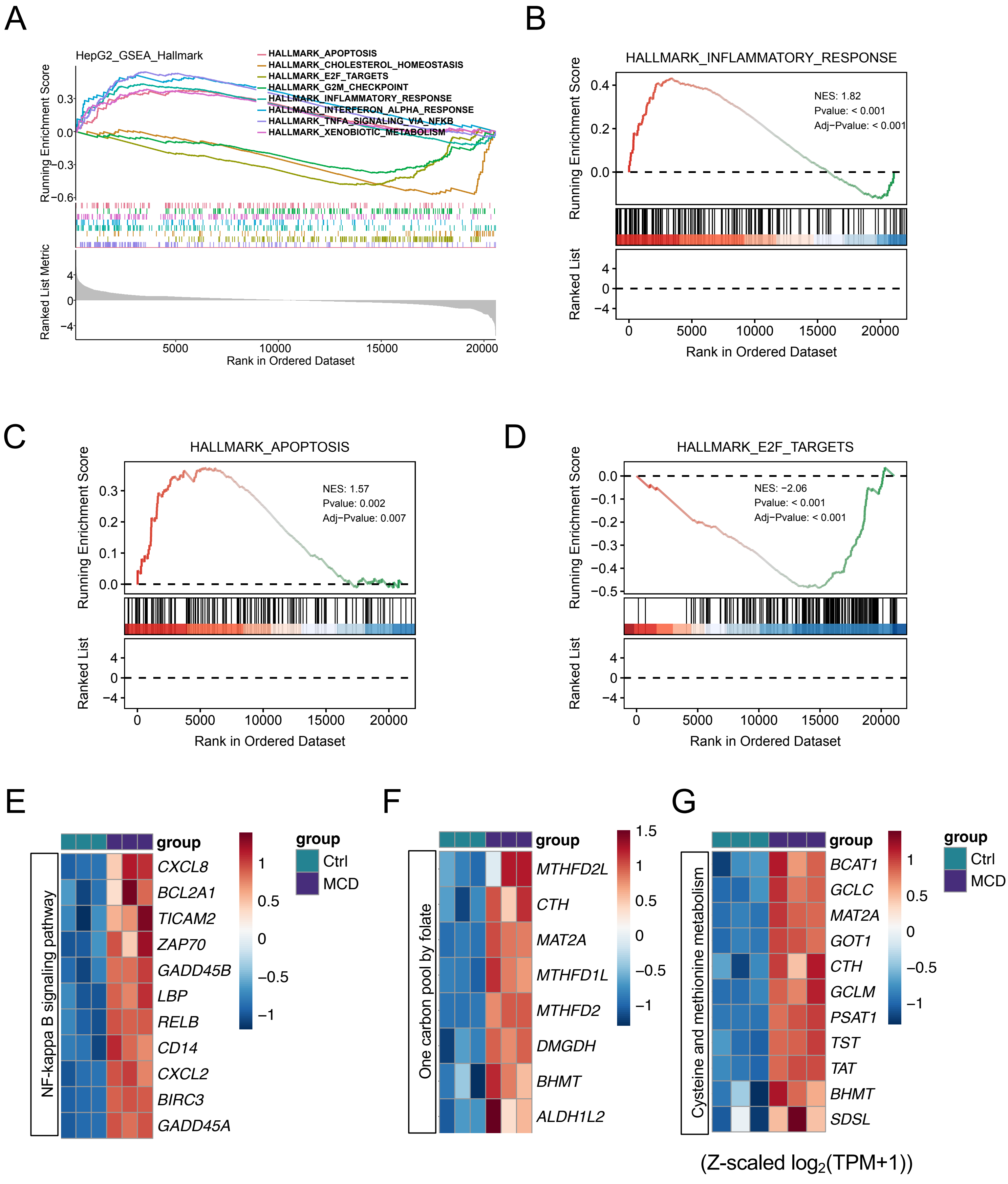

A
HepG2_GSEA_Hallmark
HALLMARK_APOPTOSIS
HALLMARK_CHOLESTEROL_HOMEOSTASIS
HALLMARK_E2F_TARGETS
HALLMARK_G2M_CHECKPOINT
HALLMARK_INFLAMMATORY_RESPONSE
HALLMARK_INTERFERON_ALPHA_RESPONSE
HALLMARK_TNFA_SIGNALING_VIA_NFKB
HALLMARK_XENOBIOTIC_METABOLISM
Running Enrichment Score
Ranked List Metric
Rank in Ordered Dataset
B
HALLMARK_INFLAMMATORY_RESPONSE
NES: 1.82
Pvalue: < 0.001
Adj−Pvalue: < 0.001
Running Enrichment Score
Ranked List
Rank in Ordered Dataset
C
HALLMARK_APOPTOSIS
NES: 1.57
Pvalue: 0.002
Adj−Pvalue: 0.007
Running Enrichment Score
Ranked List
Rank in Ordered Dataset
D
HALLMARK_E2F_TARGETS
NES: −2.06
Pvalue: < 0.001
Adj−Pvalue: < 0.001
Running Enrichment Score
Ranked List
Rank in Ordered Dataset
E
NF-kappa B signaling pathway
group
CXCL8
BCL2A1
TICAM2
ZAP70
GADD45B
LBP
RELB
CD14
CXCL2
BIRC3
GADD45A
group
Ctrl
MCD
F
One carbon pool by folate
group
MTHFD2L
CTH
MAT2A
MTHFD1L
MTHFD2
DMGDH
BHMT
ALDH1L2
G
Cysteine and methionine metabolism
group
BCAT1
GCLC
MAT2A
GOT1
CTH
GCLM
PSAT1
TST
TAT
BHMT
SDSL
group
Ctrl
MCD
(Z-scaled log2(TPM+1))

Figure S3

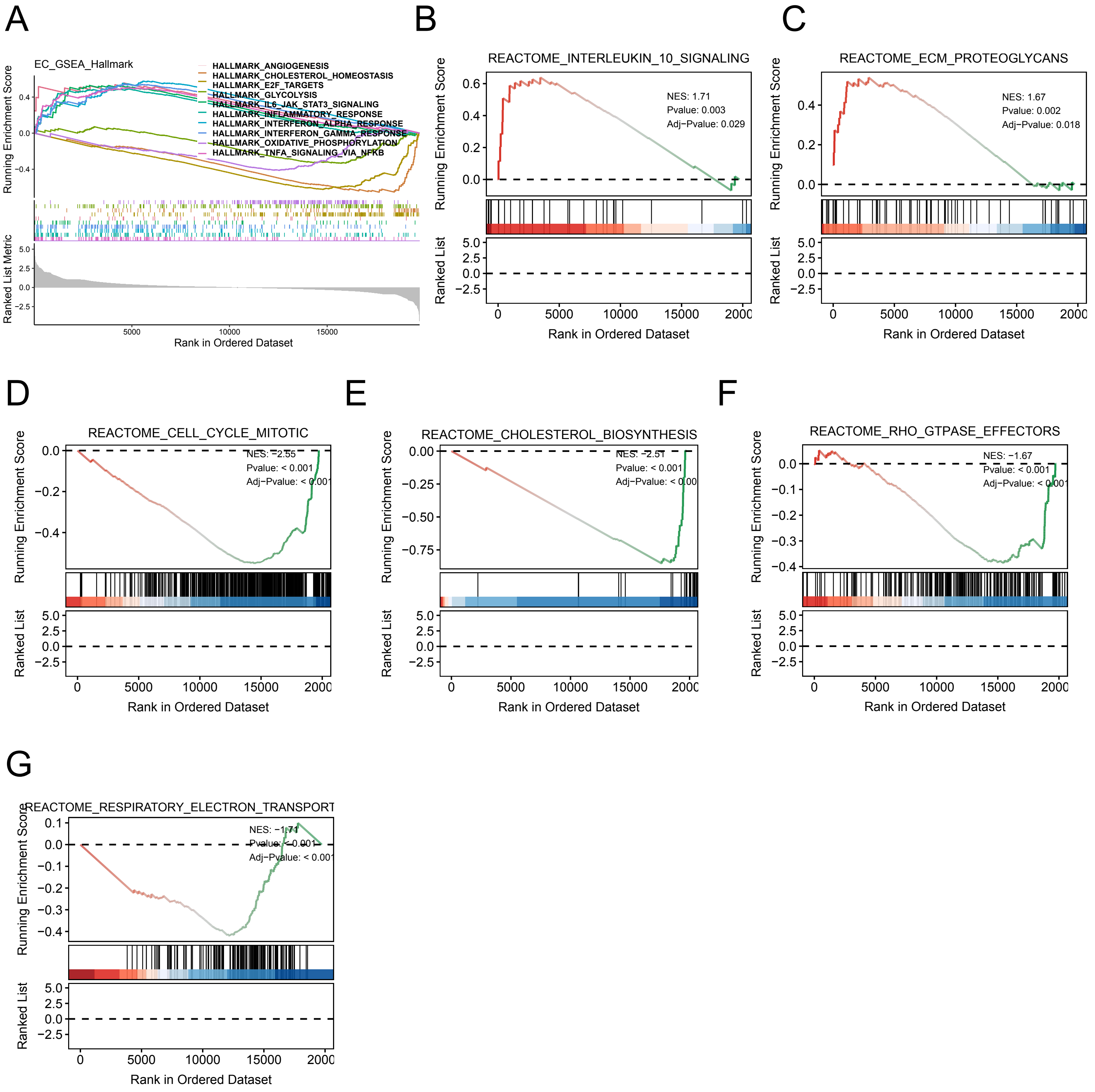
A
EC_GSEA_Hallmark
HALLMARK_ANGIOGENESIS
HALLMARK_CHOLESTEROL_HOMEOSTASIS
HALLMARK_E2F_TARGETS
HALLMARK_GLYCOLYSIS
HALLMARK_IL6_JAK_STAT3_SIGNALING
HALLMARK_INFLAMMATORY_RESPONSE
HALLMARK_INTERFERON_ALPHA_RESPONSE
HALLMARK_INTERFERON_GAMMA_RESPONSE
HALLMARK_OXIDATIVE_PHOSPHORYLATION
HALLMARK_TNFA_SIGNALING_VIA_NFKB
Running Enrichment Score
Ranked List Metric
Rank in Ordered Dataset
B
REACTOME_INTERLEUKIN_10_SIGNALING
NES: 1.71
Pvalue: 0.003
Adj−Pvalue: 0.029
C
REACTOME_ECM_PROTEOGLYCANS
NES: 1.67
Pvalue: 0.002
Adj−Pvalue: 0.018
D
REACTOME_CELL_CYCLE_MITOTIC
NES: −2.55
Pvalue: < 0.001
Adj−Pvalue: < 0.001
E
REACTOME_CHOLESTEROL_BIOSYNTHESIS
NES: −2.51
Pvalue: < 0.001
F
REACTOME_RHO_GTPASE_EFFECTORS
NES: −1.67
Pvalue: < 0.001
Adj−Pvalue: < 0.001
G
REACTOME_RESPIRATORY_ELECTRON_TRANSPORT
NES: −1.71
Pvalue: < 0.001
Adj−Pvalue: < 0.001
Running Enrichment Score
Ranked List
Rank in Ordered Dataset

Figure S4

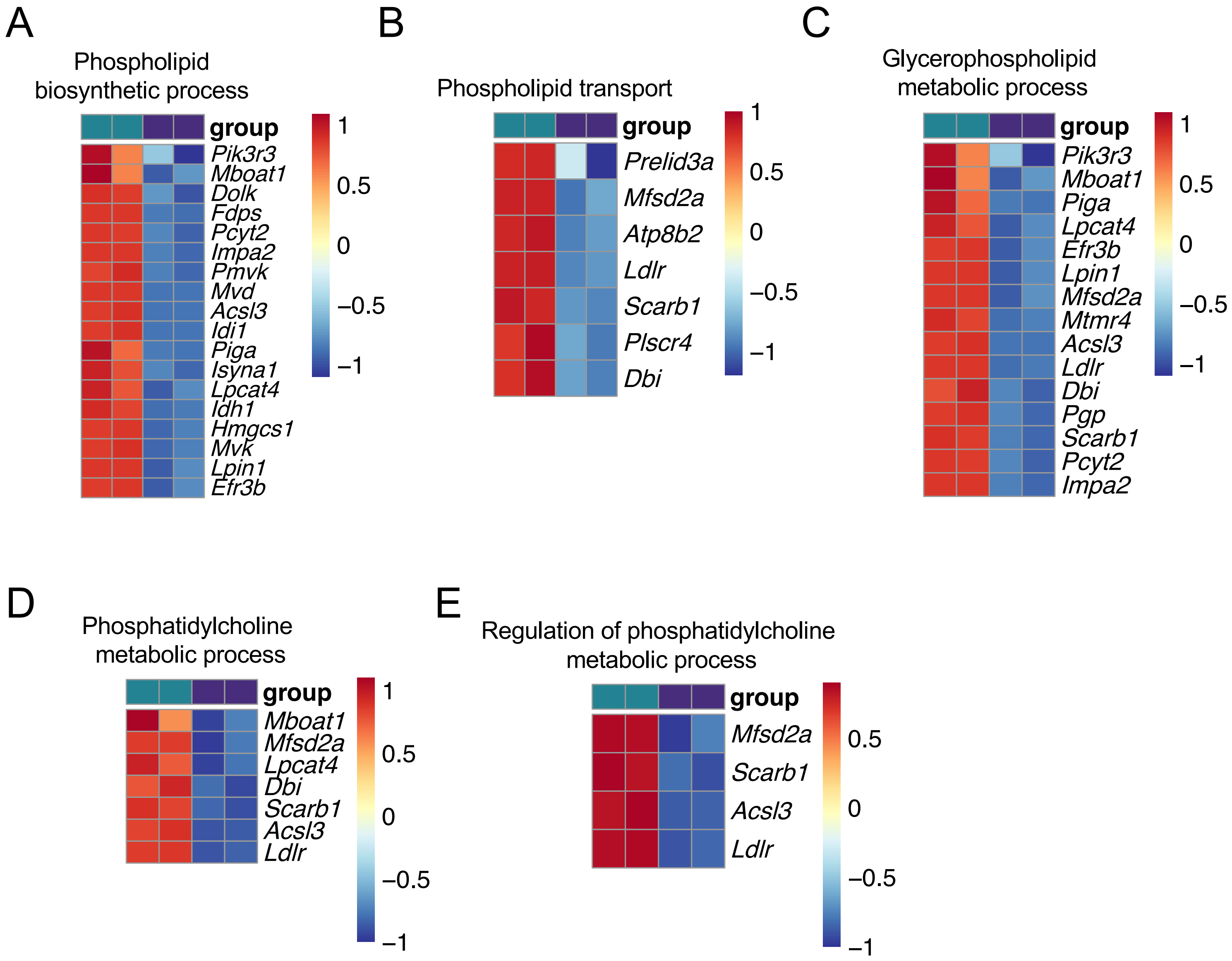
A
Phospholipid biosynthetic process
group
Pik3r3
Mboat1
Dolk
Fdps
Pcyt2
Impa2
Pmvk
Mvd
Acsl3
Idi1
Piga
Isyna1
Lpcat4
Idh1
Hmgcs1
Mvk
Lpin1
Efr3b
1
0.5
0
−0.5
−1
B
Phospholipid transport
group
Prelid3a
Mfsd2a
Atp8b2
Ldlr
Scarb1
Plscr4
Dbi
1
0.5
0
−0.5
−1
C
Glycerophospholipid metabolic process
group
Pik3r3
Mboat1
Piga
Lpcat4
Efr3b
Lpin1
Mfsd2a
Mtmr4
Acsl3
Ldlr
Dbi
Pgp
Scarb1
Pcyt2
Impa2
1
0.5
0
−0.5
−1
D
Phosphatidylcholine metabolic process
group
Mboat1
Mfsd2a
Lpcat4
Dbi
Scarb1
Acsl3
Ldlr
1
0.5
0
−0.5
−1
E
Regulation of phosphatidylcholine metabolic process
group
Mfsd2a
Scarb1
Acsl3
Ldlr
0.5
0
−0.5
−1

Figure S5

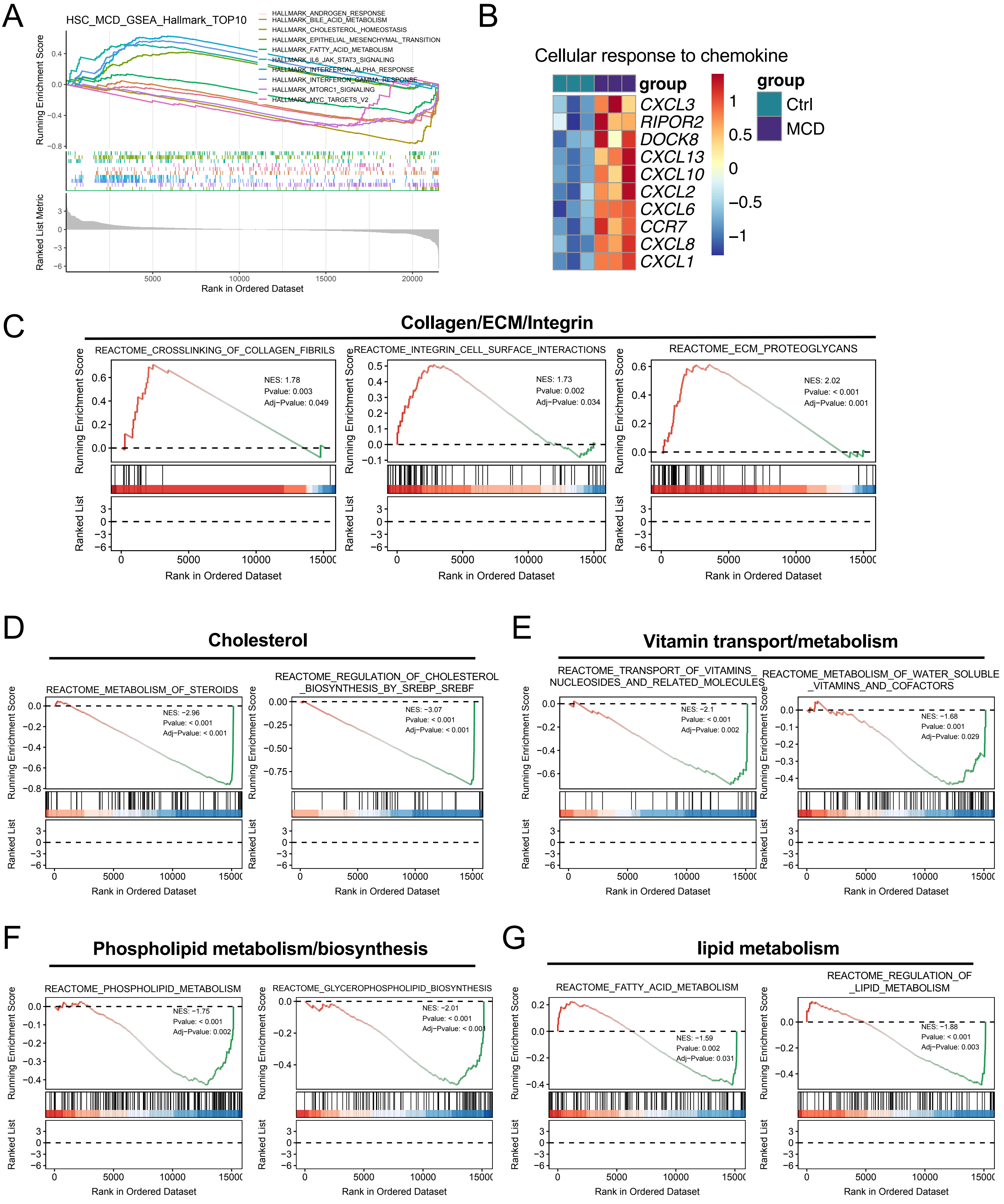
A
HSC_MCD_GSEA_Hallmark_TOP10
HALLMARK_ANDROGEN_RESPONSE
HALLMARK_BILE_ACID_METABOLISM
HALLMARK_CHOLESTEROL_HOMEOSTASIS
HALLMARK_EPITHELIAL_MESENCHYMAL_TRANSITION
HALLMARK_FATTY_ACID_METABOLISM
HALLMARK_IL6_JAK_STAT3_SIGNALING
HALLMARK_INTERFERON_ALPHA_RESPONSE
HALLMARK_INTERFERON_GAMMA_RESPONSE
HALLMARK_MTORC1_SIGNALING
HALLMARK_MYC_TARGETS_V2
Running Enrichment Score
Ranked List Metric
Rank in Ordered Dataset
B
Cellular response to chemokine
group
CXCL3
RIPOR2
DOCK8
CXCL13
CXCL10
CXCL2
CXCL6
CCR7
CXCL8
CXCL1
group
Ctrl
MCD
C
Collagen/ECM/Integrin
REACTOME_CROSSLINKING_OF_COLLAGEN_FIBRILS
NES: 1.78
Pvalue: 0.003
Adj−Pvalue: 0.049
REACTOME_INTEGRIN_CELL_SURFACE_INTERACTIONS
NES: 1.73
Pvalue: 0.002
Adj−Pvalue: 0.034
REACTOME_ECM_PROTEOGLYCANS
NES: 2.02
Pvalue: < 0.001
Adj−Pvalue: 0.001
D
Cholesterol
REACTOME_METABOLISM_OF_STEROIDS
NES: −2.96
Pvalue: < 0.001
Adj−Pvalue: < 0.001
REACTOME_REGULATION_OF_CHOLESTEROL_BIOSYNTHESIS_BY_SREBP_SREBF
NES: −3.07
Pvalue: < 0.001
Adj−Pvalue: < 0.001
E
Vitamin transport/metabolism
REACTOME_TRANSPORT_OF_VITAMINS_NUCLEOSIDES_AND_RELATED_MOLECULES
NES: −2.1
Pvalue: < 0.001
Adj−Pvalue: 0.002
REACTOME_METABOLISM_OF_WATER_SOLUBLE_VITAMINS_AND_COFACTORS
NES: −1.68
Pvalue: 0.001
Adj−Pvalue: 0.029
F
Phospholipid metabolism/biosynthesis
REACTOME_PHOSPHOLIPID_METABOLISM
NES: −1.75
Pvalue: < 0.001
Adj−Pvalue: 0.002
REACTOME_GLYCEROPHOSPHOLIPID_BIOSYNTHESIS
NES: −2.01
Pvalue: < 0.001
Adj−Pvalue: < 0.001
G
lipid metabolism
REACTOME_FATTY_ACID_METABOLISM
NES: −1.59
Pvalue: 0.002
Adj−Pvalue: 0.031
REACTOME_REGULATION_OF_LIPID_METABOLISM
NES: −1.88
Pvalue: < 0.001
Adj−Pvalue: 0.003
Running Enrichment Score
Ranked List
Rank in Ordered Dataset
